# Hepatic Safety Profile of Atomoxetine and Methylphenidate in Patients with ADHD: Disproportionality Analysis Using EudraVigilance Database Data

**DOI:** 10.3390/ph19071122

**Published:** 2026-07-21

**Authors:** Raffaella Di Napoli, Ludovica Vittoria Laino, Concetta Rafaniello, Luigi Di Costanzo, Maria Giuseppa Sullo, Cristina Scavone, Annalisa Capuano

**Affiliations:** 1 Department of Experimental Medicine, University of Campania “L. Vanvitelli”, 80138 Naples, Italy; 2 Campania Regional Centre for Pharmacovigilance and Pharmacoepidemiology, 80138 Naples, Italy; 3 Azienda Ospedaliera Universitaria Luigi Vanvitelli, 80138 Naples, Italy; 4 Department of Life Science, Health, and Health Professions, Link Campus University, 00165 Roma, Italy

**Keywords:** hepatobiliary disorders, atomoxetine, methylphenidate, ADHD, disproportionality analysis, adverse event, pooled analysis, EudraVigilance, pharmacovigilance analysis

## Abstract

**Background**: Hepatotoxicity induced by atomoxetine (ATX) and methylphenidate (MPH) when used to treat ADHD is a rare but potentially serious complication. This study aims to describe the hepatic adverse drug reactions (ADRs) reported for ATX and MPH by analysing data from the EudraVigilance database. **Methods**: Individual case safety reports (ICSRs) listing ATX and/or MPH as suspected drugs and reporting at least one adverse event (AE) within the ‘hepatobiliary disorders’ system organ class (SOC) were extracted for the period from 1 January 2012 to 20 May 2025. Descriptive and disproportionality analyses were then performed. **Results**: During the study period, 421 ICSRs reporting AEs classified under the “hepatobiliary disorders” SOC and involving ATX and/or MPH as suspected drugs were retrieved (ATX, N = 232; MPH, N = 181). Most cases involved adult (N = 261) and female (N = 222) patients. The majority of reports were classified as serious (N = 349). Overall, 375 AEs were identified. Drug-induced liver injury (DILI) was the most frequently reported AE (N = 103 ATX; N = 47 MPH), followed by hepatitis (N = 20 ATX; N = 9 MPH) and jaundice (N = 15 ATX; N = 20 MPH). The disproportionality analysis, based on a head-to-head comparison, showed a higher reporting frequency of hepatobiliary disorders for ATX compared to MPH (ROR 2.41 [95%CI 2.11–3.11]). Specifically, ATX was associated with significantly higher reporting frequencies than MPH for the AEs of DILI, hepatitis, and jaundice (6.42 [4.45–9.06]; 6.48 [2.95–14.24]; and 2.19 [1.12–4.27], respectively). **Conclusions**: This analysis, based on the EudraVigilance database, suggests that both drugs are associated with hepatobiliary adverse drug reactions, with a higher overall reporting frequency observed for ATX.

## 1. Introduction

Attention deficit/hyperactivity disorder (ADHD) is a neurodevelopmental disorder that is commonly diagnosed during childhood. It negatively impacts functioning, especially in the academic and social domains, and has a chronic course [1]. Comorbidities are frequent, mainly psychiatric and/or development disorders [2]. In addition, children suffering from ADHD have a higher chance of presenting adverse clinical outcomes throughout their lifespan, such as accidental injuries, suicide, obesity, smoking, excess alcohol use, and coronary heart disease [3]. Worldwide, ADHD affects approximately 5–10% of children/adolescents and 2.5% of adults, with higher rates among males than females (11–17% vs. 7–8%) [4,5]. There are three main ADHD subtypes, which are based on the prevalence of inattention or hyperactivity–impulsivity symptoms according to the Diagnostic and Statistical Manual of Mental Disorders, Fifth Edition, Text Revision (DSM-5-TR): predominantly inattentive type (ADHD-I), predominantly hyperactive–impulsive type (ADHD-H), and combined type (ADHD-C) [6,7]. The pharmacological treatment of ADHD includes nonstimulants, mainly atomoxetine (ATX), and psychostimulants, such as amphetamines and methylphenidate (MPH). Worldwide, MPH and ATX are the most common medications used to treat ADHD [8]. ATX is a selective inhibitor of the norepinephrine transporter (NET), increasing norepinephrine (noradrenaline) levels in the prefrontal cortex [9]. MPH is instead a reuptake transporter for dopamine and norepinephrine inhibitor that increases synaptic levels of both catecholamines, especially dopamine in striatal and cortical circuits. During treatment with MPH, the dopaminergic and noradrenergic tone is enhanced, which correlates with improved attention, impulse control, and executive functioning [10,11]. As a chronic condition, ADHD requires long-term pharmacological treatment; thus, the monitoring of ATX and MPH are of great concern to healthcare stakeholders. Apart from cardiovascular and psychiatric concerns [12,13,14,15,16], both drugs have also been associated with hepatotoxicity in children [17,18]. Based on the available literature data, ATX- and MPH-induced hepatotoxicity remains a rare but potentially serious complication. As rare adverse drug reactions (ADRs), such as hepatotoxicity, may not emerge during pre-marketing clinical trials, post-marketing pharmacovigilance systems utilising real-world data from spontaneous reporting databases are essential for detecting and characterising these events in heterogeneous paediatric populations undergoing long-term treatment [19,20].

The aim of this paper is to characterise and compare reports of hepatobiliary adverse drug reactions (ADRs) associated with atomoxetine (ATX) and methylphenidate (MPH) in the EudraVigilance (EV) database.

The primary objective was to evaluate whether hepatobiliary ADRs were reported more frequently for one drug than the other. Reporting odds ratios (RORs) and 95% confidence intervals (CIs) were therefore calculated for hepatobiliary adverse reactions overall and for selected preferred terms. Further analyses were performed, stratified by sex, age group, and psychiatric co-medication, to investigate reporting patterns within each drug.

## 2. Results

### 2.1. Descriptive Analysis of Cases

**Figure 1:**
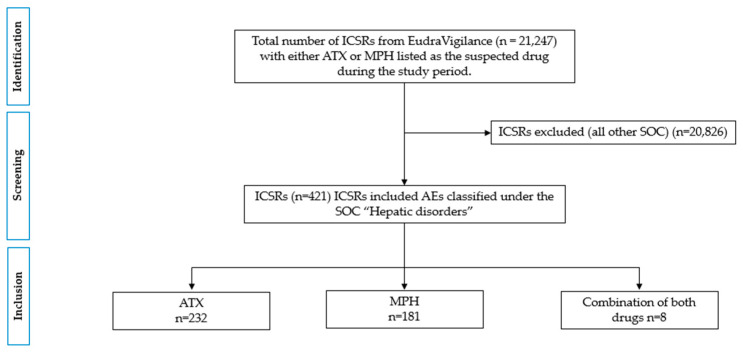
Flow chart showing individual case safety reports (ICSRs) retrieved from the EudraVigilance database that reported atomoxetine (ATX) and/or methylphenidate (MPH) as the suspected drugs. The focus was on hepatobiliary disorders (SOC).

During the study period, a total of 21,247 ICSRs in which ATX and/or MPH were reported as suspected drugs were retrieved from the EV database (Figure 1). Of these, 421 ICSRs included adverse events (AEs) classified under the system organ class (SOC) “hepatobiliary disorders”. Specifically, 232 cases involved ATX, 181 involved MPH, and eight were associated with the combination of both drugs. In terms of age distribution, 124 cases involved patients aged 0–17 years, 261 cases involved patients aged 18 years or older, and 36 cases did not specify the patient’s age.

#### 2.1.1. Descriptive Analysis of Patients Under 18 Years Old

A total of 124 ICSRs, covering 142 preferred terms (PTs), were identified in this age group, of which 91 involved MPH as the suspected drug and 33 cases involved ATX. There were no reports relating to the combination of these drugs. Male patients accounted for most reports overall, representing 60.6% of ATX cases and 68.1% of MPH cases. The majority of ICSRs (94.4%) were submitted by healthcare professionals (HCPs). In terms of the country of origin for regulatory purposes, reports relating to ATX predominantly originated from countries outside the European economic area (EEA) (N = 22; 66.7%), whereas reports relating to MPH predominantly originated from EEA countries (N = 62; 68.1%). More than 50% of ICSRs related to ATX and MPH reported only one suspected drug, and no concomitant drugs were reported in 79% of cases. The main demographic and clinical characteristics of these cases are summarised in Table 1.

Table 2 shows the AEs reported in this age group. Of the 142 PTs selected as potentially closely related to the suspected drugs, 99 were related to MPH and 43 to ATX (Table 2). Most of these AEs were classified as serious: 100% were ATX-related AEs and 84.8% were MPH-related AEs. The most reported seriousness criterion for both drugs was “other medically important condition”. Regarding the clinical outcome, ATX-related AEs were more frequently reported as recovered/resolved (46.5%), whereas the outcome was most often reported as unknown in the MPH group (34.3%).

#### 2.1.2. Descriptive Analysis of Patients over 18 Years Old

A total of 261 ICSRs, covering 197 PTs, were identified in this age group, of which 183 reported ATX, 70 reported MPH, and eight reported the combination. Female patients represented most reports in the ATX (84.7%) and combination (87.5%) groups, whereas the MPH group was predominantly associated with male patients (54.3%). Overall, most reports (91.6%) were submitted by HCPs. ICSRs relating to ATX (97.3%) and combination therapy (87.5%) predominantly originated from non-EEA countries, whereas reports relating to MPH were more frequently reported from EEA countries (54.3%). Most ICSRs in this age group (63.2%) listed five or more suspected drugs. Concomitant drugs were not reported in 53.3% of cases. The demographic and clinical characteristics are detailed in Table 1. In terms of AEs reported in this age group, 197 PTs were selected as potentially closely related to the suspected drugs. Of these, 127 were related to ATX, 62 to MPH, and eight to the combination group (Table 2). Most of these AEs were classified as serious (94.9%), mainly reported as “results in death” for ATX (66.9%) and “other medically important condition” for MPH (54.8%). Regarding clinical outcomes, ATX-related AEs were most often reported as fatal (66.9%), while the outcome was most frequently reported as unknown in the MPH group (51.6%). Among the fatal adult ATX reports, only three cases were coded with PTs indicative of suicide- or overdose-related events, including one overdose, one prescribed overdose, and one completed suicide.

#### 2.1.3. Descriptive Analysis of Patients Ages Not Specified

A total of 36 ICSRs, covering 36 PTs, were included in this category, comprising 16 ATX-related and 20 MPH-related ICSRs. Overall, male patients accounted for most reports (47.2%), and most ICSRs (66.7%) were submitted by HCPs. The non-EEA region was the most represented source of reports (52.8%). Overall, the majority of ICSRs (63.9%) reported only one suspected drug, and 80.6% of cases had no concomitant drug listed. Detailed demographic and clinical characteristics are presented in Table 1. Almost 97% of reported PTs were classified as serious, mainly as “other medically important condition”. The clinical outcome was unknown for most AEs (66.7%) (Table 2).

### 2.2. Description of Hepatobiliary Disorders AEs

In total, 375 PTs were selected from the AEs reported under the SOC ‘hepatobiliary disorders’, which were deemed to be potentially closely related to the suspected drugs, as shown in Table 3. These PTs were classified as follows: “hepatic failure, fibrosis, cirrhosis, and other liver damage-related conditions” (62.4%); “hepatitis, non-infectious” (14.4%); and “liver-related investigations, signs and symptoms” (23.2%). The most frequently reported AE in the first category was drug-induced liver injury (DILI), accounting for 156 cases. Of these, 66.0% were associated with ATX, 30.1% with MPH, and 3.8% with both drugs combined. The most commonly reported AE in the second group was hepatitis, of which 66% attributed to ATX, 30% to MPH, and 3.3% to the combination. In the third category, the most frequently reported AE was jaundice, of which 42.9% related to ATX, and 57.1% to MPH. PTs not considered to be closely related to the suspected drugs and instead were related to independent hepatic diseases are reported in the Supplementary Materials (Table S1).

### 2.3. Results of Disproportionality Analyses

**Figure 2:**
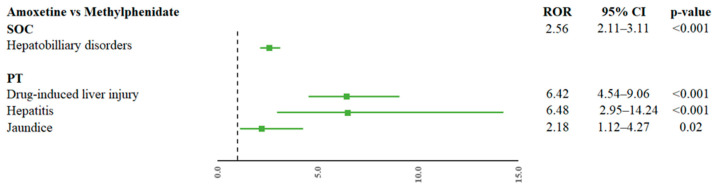
Reporting odds ratios (RORs) for the system organ class (SOC) hepatobiliary disorders and the preferred terms (PTs) “drug-induced liver injury”, “hepatitis”, and “jaundice”, comparing atomoxetine and methylphenidate. RORs and their 95% confidence intervals (CIs) were calculated and presented in two ways: graphically, as a forest plot, and numerically. In the forest plot, statistically significant results are highlighted in green. A *p*-value of less than 0.05 was considered statistically significant.

As shown in Figure 2, the primary analysis revealed a statistically significant higher disproportionate reporting for ATX than MPH across all SOC hepatobiliary disorders (ROR = 2.41, 95% CI: 2.11–3.11). Specifically, ATX was associated with significantly higher disproportionate reporting ratio than MPH for the PTs of DILI, hepatitis, and jaundice (ROR = 6.42, 95% CI: 4.45–9.06; ROR = 6.48, 95% CI: 2.95–14.24; and ROR = 2.19, 95% CI: 1.12–4.27, respectively).

**Figure 3:**
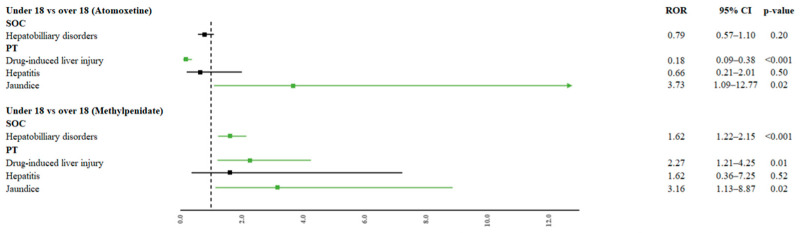
Reporting odds ratios (RORs) for the system organ class (SOC) hepatobiliary disorders and the preferred terms (PTs) “drug-induced liver injury”, “hepatitis”, and “jaundice”, comparing atomoxetine and methylphenidate in patients under 18 years of age versus patients aged 18 years and over. RORs and their 95% confidence intervals (CIs) were calculated and presented in two ways: graphically, as a forest plot, and numerically. In the forest plot, non-significant results are shown as black squares with horizontal lines, while statistically significant results are highlighted in green. A *p*-value of less than 0.05 was considered statistically significant.

Figure 3 shows that, when the data were stratified by age, disproportionate analysis revealed lower reporting of DILI in younger patients (under 18 years old) than in adult ATX users (ROR = 0.18, 95% CI: 0.09–0.38). It also revealed higher reporting of jaundice in younger patients than in adults (ROR = 3.74, 95% CI: 1.09–12.77). No statistically significant differences were observed between the two age groups for the SOC hepatobiliary disorders and PT hepatitis.

For MPH, higher disproportionate reporting of the SOC hepatobiliary disorders and the PTs of DILI and jaundice was observed in patients < 18 years compared to those >18 years (hepatobiliary disorders—ROR = 1.62, 95% CI: 1.22–2.15; DILI—ROR = 2.27, 95% CI: 1.21–4.25; jaundice—ROR = 3.16, 95% CI: 1.13–8.87; Figure 3). No statistically significant difference was observed between the two age groups for the PT hepatitis.

**Figure 4:**
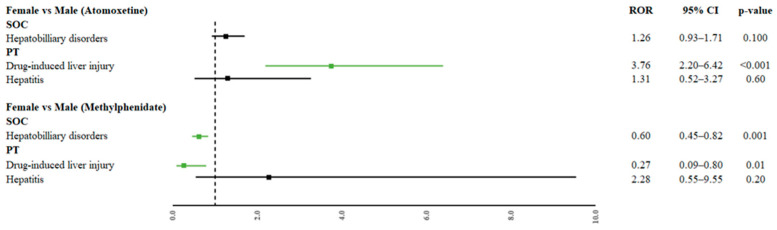
Reporting odds ratios (RORs) for the system organ class (SOC) hepatobiliary disorders and the preferred terms (PTs) “drug-induced liver injury”, “hepatitis”, and “jaundice”, comparing atomoxetine and methylphenidate between male and female patients. RORs and their 95% confidence intervals (CIs) were calculated and presented in two ways: graphically, as a forest plot, and numerically. In the forest plot, non-significant results are shown as black squares with horizontal lines, while statistically significant results are highlighted in green. A *p*-value of less than 0.05 was considered statistically significant.

As shown in Figure 4, the analysis stratified by sex revealed that ATX was associated with higher disproportionate reporting DILI in female patients than in male patients (ROR = 3.76, 95% CI: 2.20–6.42). No statistically significant differences were observed between sexes for the SOC hepatobiliary disorders and the PT hepatitis. Conversely, a lower disproportionate reporting was observed in females than in males in the MPH group for both SOC hepatobiliary disorders and PT DILI (SOC—ROR = 0.61, 95% CI: 0.45–0.82; DILI—ROR = 0.27, 95% CI: 0.09–0.80; Figure 4). Similarly, no statistically significant sex-related differences were observed for PT hepatitis.

**Figure 5:**
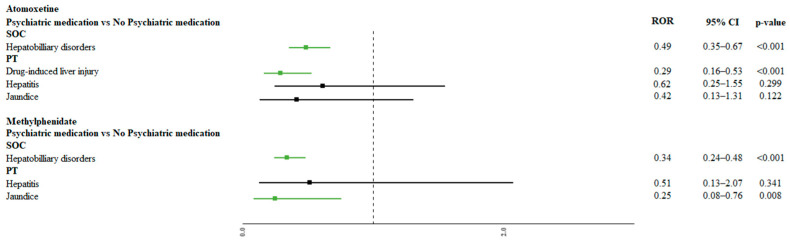
Reporting odds ratios (RORs) for the system organ class (SOC) hepatobiliary disorders and the preferred terms (PTs) “drug-induced liver injury”, “hepatitis”, and “jaundice”, comparing atomoxetine and methylphenidate between concomitant psychiatric medications and no concomitant psychiatric medications. RORs and their 95% confidence intervals (CIs) were calculated and presented in two ways: graphically, as a forest plot, and numerically. In the forest plot, non-significant results are shown as black squares with horizontal lines, while statistically significant results are highlighted in green. A *p*-value of less than 0.05 was considered statistically significant.

As shown in Figure 5, the presence or absence of concomitant psychiatric medications was used to stratify the analysis. This indicated that ATX was associated with lower disproportionate reporting of hepatobiliary disorders and PT DILI in patients receiving psychiatric medications compared to those not receiving them (ROR = 0.49, 95% CI: 0.35–0.67 and ROR = 0.29, 95% CI: 0.16–0.53, respectively). No statistically significant difference was observed between these two groups for the PTs of hepatitis and jaundice. Similarly, lower disproportionate reporting was observed in the MPH group for both SOC hepatobiliary disorders (ROR = 0.34, 95% CI: 0.24–0.48) and PT jaundice (ROR = 0.25, 95% CI: 0.08–0.76) in patients receiving psychiatric medications compared to those not receiving them (Figure 5). No statistically significant difference was observed between these two groups for the PT hepatitis.

## 3. Discussion

ADHD is a neurodevelopmental condition commonly treated with pharmacological agents, including stimulant (e.g., MPH, amphetamines) and nonstimulant medications (e.g., ATX, guanfacine, clonidine). Though these medications are effective in reducing core ADHD symptoms, they are associated with potential clinical adverse outcomes [1,10,21]. Even though clinically significant hepatotoxicity seems to be rare, ATX can be associated with a small but meaningful risk of liver injury, which can vary from minor, transient, and asymptomatic elevations in serum aminotransferase levels to clinically apparent hepatitis that can be prolonged and even fatal [22].

We carried out a pharmacovigilance study based on data from the EV database with the aim to better characterise the hepatic safety profile of ATX and MPH. During the study period, 421 ICSRs reporting cases of hepatic ADRs were retrieved from the EV, of which 232 related to ATX and 181 related to MPH. Almost 30% of these ICSRs concerned paediatric patients; female patients accounted for the majority of ICSRs overall for both ATX and MPH, though males were mostly represented among patients aged < 18 years. The majority of ICSRs were submitted by HCPs, and ICSRs related to ATX predominantly originated from countries outside the EEA while reports relating to MPH predominantly originated from EEA countries. A recent multinational observational study, which collected electronic health record data from five countries (Belgium, Germany, the Netherlands, Spain, the UK) within the DARWIN EU^®^ network [21], highlighted that patients who initiated any approved ADHD medication (*n* = 198.167) were predominantly male, and that the proportion of adults receiving ADHD medications ranged from 33% to 57%. The study’s results also reported that common comorbidities prior to medication initiation included depression, anxiety, and asthma. Similarly, other studies that analysed the trend in medication use for ADHD among children and adolescents reported a higher proportion of male patients receiving mediations compared to females, with male-to-female ratios ranging from 4.5:1 [23] to 2.7:1, 3.6:1, and 3.0:1 [24] across different EU countries. Some authors suggested that this difference could be related to a lower rating on core symptoms of ADHD among females, which reflects a general delay in recognising ADHD in this population [25,26].

Our results showed a higher disproportionate reporting of hepatobiliary disorders for ATX than MPH at the SOC level. The stratified analysis further supported this finding, demonstrating significantly higher disproportionate reporting for ATX than MPH for the preferred terms of DILI, hepatitis, and jaundice. These AEs have been previously reported; for instance, Bangs et al. reported that, during a 4-year post-marketing surveillance on a population of 4.3 million patients, ATX exposure was associated with three cases of reversible hepatitis [27]. Data from a study based on data from the Italian ADHD National Registry highlighted that, out of 68 paediatric patients receiving the drug, two patients experienced severe hepatic events after 7 and 10 months at unspecified doses [28]. For most drugs, the mechanism underlying the occurrence of hepatotoxicity is idiosyncratic, which implies that DILI develops in only a small proportion of subjects as a consequence of the interaction between genetic and environmental risk factors, making the toxicity itself unpredictable [17]. For this reason, drug-induced hepatotoxicity represents a major problem in all phases of clinical drug development and the most frequent cause of post-marketing warnings and withdrawals [29].

In most of cases, hepatotoxicity is associated with the production of reactive oxygen species (ROS) when drugs are metabolised by hepatic cytochrome P450 enzymes. Excessive ROS production can lead to oxidative stress, mitochondrial dysfunction, lipid peroxidation, and damage to cellular macromolecules. Ultimately, this can result in hepatocyte death via necrosis or apoptosis [15,30,31,32].

ATX is primarily metabolised by cytochrome P450 enzymes, particularly CYP2D6. Studies of its biotransformation in human liver microsomes in vitro have identified 4-hydroxy-ATX (4-hydroxy-ATX) and its glucuronide conjugate as the primary metabolites of phase I and phase II metabolism [33]. The formation of hydroxycarboxy-ATX suggests oxidation through an aldehyde intermediate, a pathway that is potentially associated with hepatotoxicity [34,35]. Consistent with this mechanism, experimental studies in human cell models have demonstrated that exposure to ATX increases ROS levels in the cytosol and mitochondria, disrupts the mitochondrial membrane potential, and impairs autophagy.

Unlike ATX, MPH metabolism is largely independent of cytochrome P450. The main elimination pathway for MPH involves rapid enzymatic hydrolysis catalysed by the hepatic carboxylesterase CES1A1 [36]. This process is stereoselective, degrading the l-threo enantiomer faster than the d-threo enantiomer. The l-threo enantiomer is responsible for the pharmacological activity. This process leads to the formation of ritalinic acid, a pharmacologically inactive metabolite that is subsequently eliminated via the kidneys.

The absence of significant CYP450 involvement reduces the formation of reactive metabolites and ROS, which are mechanisms known to underlie many forms of drug-induced hepatotoxicity. Furthermore, evidence from the literature suggests that MPH hydrolysis may also occur in extrahepatic sites, such as the blood or gastrointestinal tract. This process may be mediated by non-enzymatic mechanisms or bacterial flora and is potentially dependent on pH. Overall, these pharmacokinetic characteristics may explain why hepatotoxic events are reported less frequently with MPH than with ATX [37].

Based on this data on MPH-induced hepatotoxicity are limited. Valbom Gonçalves D et al. described the case of a 12-year-old female patient with ADHD who had been receiving MPH treatment since the age of eight and developed a marked elevation in transaminases and gamma-glutamyl transferase [38]. Jason J. Lewis et al. reported another case involving a 57-year-old Caucasian man with a history of liver transplantation for chronic hepatitis C. He developed acute liver enzyme elevation after starting a long-acting MPH derivative. The liver biopsy findings, together with the presence of autoimmune markers, were consistent with a diagnosis of autoimmune hepatitis [39]. Mehta et al. reported the case of a 19-year-old African American woman with elevated transaminase levels who had recently used an abnormal dose of intravenous MPH. Following supportive care and complete discontinuation of the drug, her levels gradually normalised. Subsequent re-administration of the drug again triggered an increase in liver enzymes, providing further evidence of MPH’s direct hepatotoxic effect [40]. A study in rats comparing MPH alone versus MPH combined with curcumin found that rats receiving MPH alone exhibited elevated liver parameters [41].

Therefore, it should be noted that MPH hepatotoxicity has occurred in specific circumstances, such as abnormal dose or underlying liver disease, as evidenced by these isolated cases and preclinical studies.

Our findings reveal a particularly striking result: the relatively high proportion of fatal hepatobiliary cases reported in connection with ATX must be interpreted with caution. Indeed, in spontaneous reporting systems, the designation of a fatal outcome does not necessarily imply a causal relationship with the suspected drug. Events may be temporally associated but determined by alternative clinical factors [42,43]. Fatal cases may also be due to underlying diseases or other illnesses or circumstances unrelated to ATX-induced liver injury. Furthermore, some reports may represent intentional overdoses or suicide attempts, in which hepatic injury may have occurred as a secondary event or been incompletely characterised.

Our analysis identified only three fatal cases coded with PTs suggestive of these circumstances (one *overdose*, one *prescribed overdose*, and one *completed suicide*). Therefore, within our dataset, fatal outcomes do not appear to be predominantly explained by overdose- or suicide-related events.

Unfortunately, the limited clinical information available in spontaneous reporting databases does not allow us to verify these circumstances or distinguish idiosyncratic drug-induced liver injury from hepatic involvement in cases of overdose or terminal illness. Furthermore, an error in the classification of the outcome cannot be ruled out. These reports are also likely to be influenced by multiple sources of confounding factors. Adult patients treated with ATX often present with complex clinical profiles, including psychiatric comorbidities, substance use, and prior treatment failure. They are also frequently exposed to polypharmacy, and all these factors may independently increase the risk of serious outcomes.

Another important difference that was revealed in our research relates to age and sex. ATX-related DILI was reported more frequently in adult and female patients, whereas MPH-related DILI was more commonly reported in younger patients and males.

To date, the literature does not provide clear evidence of a specific link between DILI and sex or age for either ATX or MPH. However, pharmacovigilance studies on large patient groups suggest that differences in susceptibility to DILI according to sex and age are likely to be drug-specific [44].

In this context, a datamining study of 375 hepatotoxic drugs, based on reports from the WHO VigiBase™ database, showed a higher overall frequency of hepatic events in women. There was an increase in reports of acute liver failure in younger women, while cholestatic damage was more frequently observed in men [45].

A study examining the incidence of drug-induced liver injury (DILI) within the general Icelandic population revealed that the age-standardised incidence rate increased significantly with advancing age. However, the underlying reasons for this association remain unclear [46]. In contrast, a large study based on the Spanish Hepatotoxicity Registry and including over 600 patients found no increased risk of DILI in older individuals [47].

Lastly, a stratified analysis of the concomitant use of psychiatric medications reinforces the idea that the observed associations with hepatobiliary disorders might be more directly attributable to the analysed drugs than to confounding factors related to the patients’ clinical profiles.

### Strengths and Limitations

As it is based on data from a spontaneous reporting system, our study suffers from several limitations, including underreporting and reporting bias. It is well-known that only a small proportion of AEs are reported, and reporting is influenced by factors such as event severity, media attention, regulatory warnings, and time since marketing. Another important limitation of this study is the absence of drug exposure data. Because the total number of patients receiving ATX and MPH is unknown, incidence rates and absolute risks of adverse events cannot be estimated. Consequently, the results should be interpreted as signal-generating and hypothesis-generating rather than providing definitive measures of risk. ICSRs often lack critical information on dose, duration, comorbidities, laboratory values, and temporal relationships, limiting causal assessment. Concomitant medications and underlying diseases may contribute to reported events, and these confounders are often insufficiently documented. In addition, differences in prescribing patterns may introduce indication and channel bias. For example, certain drugs (such as second-line treatments like ATX) may be prescribed more often to patients with specific clinical characteristics or who have experienced prior treatment failure. Finally, the limited clinical detail available in many reports restricts assessment of confounding factors, patient characteristics, and the clinical context of reported adverse events. This could potentially influence the observed reporting patterns.

Importantly, the application of formal causality assessment methods (e.g., the updated RUCAM [15]) is generally not feasible or reliable due to the nature and structure of spontaneous reporting databases, such as EudraVigilance. These methods require detailed, standardised, and prospectively collected clinical data, which is usually unavailable in pharmacovigilance datasets. Therefore, attempting to apply such tools in this context could lead to inconsistent or biassed attribution of causality. For this reason, it is not possible to establish definitive proof of causality between ATX/MPH and hepatic adverse events (AEs), and the findings of our study should be interpreted as signals of potential association rather than evidence of a causal relationship.

Notwithstanding these limitations, data from the spontaneous reporting systems are highly effective for identifying rare, serious, or unexpected ADRs, particularly those not detected during pre-marketing clinical trials, such as those with an idiosyncratic underlying mechanism. In addition, spontaneous reporting systems capture data from real-world use across diverse populations, including frail populations (such as paediatrics and pregnancy women) [48,49,50,51] and patients with comorbidities, polypharmacy, and off-label use, enhancing external validity. Studies based on these data are relatively inexpensive, continuous, and allows for rapid signal generation soon after a drug enters the market. Lastly, pharmacovigilance studies are exploratory by nature and are specifically designed to detect signals through disproportionality analyses rather than to test hypotheses or infer causality. Within this framework, they are an essential first step in identifying potential safety concerns, which can then be investigated further using more robust epidemiological or clinical studies designed to establish causality.

## 4. Methodology

### 4.1. Study Design

A pharmacovigilance study was conducted using the EV database to analyse the reporting frequency of hepatobiliary disorders associated with ATX or MPH use. The study was conducted in accordance with the Reporting of a Disproportionality Analysis for Drug Safety Signal Detection Using ICSRs in Pharmacovigilance (READUS-PV) guideline [52].

### 4.2. Data Source

The EV database contains reports of suspected ADRs relating to drugs and vaccines authorised within the EEA. Managed by the European Medicines Agency (EMA) on behalf of the European Union regulatory network, the database is publicly accessible via the ADR Reports portal: https://www.adrreports.eu/en/search.html (accessed on 20 May 2025). Each report includes information on patient characteristics, the type of reporter, the country of origin (for regulatory purposes), the suspected and concomitant drugs involved, and the seriousness and outcome of the ADR. A suspected drug is defined as a medicinal product that the reporter considers to be potentially related to an adverse event. This classification indicates a suspected association rather than a confirmed causal relationship, and adverse events may also be influenced by other medications taken concurrently or patient-related factors.

ADRs recorded in the EV are coded using the Medical Dictionary for Regulatory Activities (MedDRA), an internationally standardised terminology used in regulatory settings to classify medical information, such as signs, symptoms, and diagnoses [53]. MedDRA is organised into five hierarchical levels, progressing from the most specific to the most general: Lowest Level Terms (LLTs), PT, High Level Terms (HLTs), High Level Group Terms (HLGTs) and SOC.

### 4.3. ICSRs Selection

ICSRs listing ATX and/or MPH as the suspected drug and including any AEs classified under the “hepatobiliary disorders” SOC, as defined by PTs, were extracted from the EV database for the period from 1 January 2012 to 20 May 2025. Version 27.0 of the MedDRA was used.

### 4.4. Ethics Statement

This study was based exclusively on anonymised adverse event reports obtained from the publicly accessible EudraVigilance database. No identifiable personal information was accessed and there was no direct patient involvement. Therefore, informed consent and Institutional Review Board (IRB)/Ethics Committee approval were not required.

### 4.5. Descriptive Analysis

A descriptive analysis was conducted on ICSRs that listed ATX and/or MPH as suspect drugs and included at least one PT under the hepatobiliary disorders as an AE. ICSRs were categorised into three age groups: 0–17 years, 18 years and over, and age not specified. Within each age group, cases were further divided into three subgroups based on the suspected drug: ATX, MPH, or a combination of both. The following characteristics were described for each age group: age, sex, primary source country, type of reporter, and number of suspected and concomitant drugs reported. A subsequent analysis was performed on all PTs reported in the selected ICSRs. These PTs were categorised according to the type of hepatic adverse events in “hepatic failure, fibrosis, cirrhosis, and other liver damage-related conditions”; “hepatitis, non-infectious”; and “liver-related investigations, signs and symptoms”. Using the structure of the SMQ for drug-related hepatobiliary disorders as a guiding reference, a team of pharmacologists and clinicians with consolidated experience in pharmacovigilance performed this classification, with the aim to guarantee high quality standard for the overall methodological process. Although the SMQ for drug-related hepatobiliary disorders was not formally applied, its structure served as a guiding reference.

The seriousness of hepatobiliary AEs was classified as either “serious” or “not serious”, with an event being considered “serious” if it met at least one of the following regulatory criteria: it caused or prolonged hospitalisation; it resulted in disability; it was life-threatening; it was considered another medically important condition; or it resulted in death [54]. The outcomes were categorised as follows: not recovered/not resolved, recovered/resolved, recovering/resolving, recovered/resolved with sequelae, or fatal.

All the qualitative variables were reported as numbers and percentages.

### 4.6. Disproportionality Analyses

To evaluate the reporting frequency of AEs under the “hepatobiliary disorders” SOC, the RORs and their 95% CIs were calculated for the SOC overall and for the three most reported PTs belonging to the previously categories that were potentially closely related to the suspected drugs, comparing ATX and MPH.

In addition, stratified analyses were conducted. First, the reporting frequency of the SOC and the three most frequently reported PTs were compared between male and female patients for each drug (ATX and MPH separately). A second stratification was then performed by age group, comparing reports in patients <18 years with those >18 years. This was done separately for ATX and MPH, considering both the SOC and the three most PTs reported. Lastly, the reporting frequency of the SOC and the three most frequently reported PTs was compared between ICSRs that reported psychiatric co-medication and those that did not. Psychiatric medications were identified by manual review of the concomitant medications and included antidepressants, antipsychotics, anxiolytics, mood stabilisers, hypnotics, and psychostimulants.

RORs were calculated only when at least three cases of either ATX or MPH were reported.

Data management and statistical analyses were performed using Microsoft Excel 365 and R software (version 4.2.2, R Development Core Team).

## 5. Conclusions

In conclusion, this pharmacovigilance analysis based on the EudraVigilance database suggests that both ATX and MPH are associated with hepatobiliary AEs, with a higher overall reporting frequency observed for ATX. The most reported events were DILI, hepatitis, and jaundice. Notable differences emerged according to age and sex, with ATX-related DILI reported more frequently in adults and female patients, whereas MPH-related DILI was more commonly reported in younger patients and males. Lastly, stratified analyses according to the use of concomitant psychiatric medications showed that the reporting of hepatobiliary disorders was lower among patients receiving such medications than among those not receiving them for both ATX and MPH. Although these findings suggest that concomitant psychiatric medications are unlikely to fully account for the observed associations, they do not rule out the potential influence of residual confounding factors related to psychiatric comorbidities, intentional or accidental overdoses, polypharmacy, and other patient-related factors. Therefore, the observed associations should be interpreted with caution. These findings highlight the need for further studies that include appropriate exposure data and clinical detail. The present results are insufficient to support differential therapeutic choices between these medications.

## Figures and Tables

**Table 1:** Characteristics of individual case safety reports (ICSRs) reporting system organ class (SOC) “hepatobiliary disorders” with atomoxetine, methylphenidate, and their combination, as recognised in the EudraVigilance spontaneous reporting system from 1 January 2012 to 20 May 2025.

Patients Under 18 Years	Atomoxetine	Methylphenidate	Combination	Overall
	(N = 33)	(N = 91)	(N = 0)	(N = 124)
**Age**				
1–12 Months	1 (3.0)	-	-	1 (0.8)
3–11 Years	11 (33.3)	37 (40.7)	-	48 (38.7)
12–17 Years	21 (63.6)	54 (59.3)	-	75 (60.5)
**Sex**				
Female	8 (24.2)	12 (13.2)	-	20 (16.1)
Male	20 (60.6)	62 (68.1)	-	82 (66.1)
Not Specified	5 (15.2)	17 (18.7)	-	22 (17.7)
**Source**				
Healthcare Professional	33 (100)	84 (92.3)	-	117 (94.4)
Non-Healthcare Professional	-	7 (7.7)	-	7 (5.6)
**Country**				
European Economic Area	11 (33.3)	62 (68.1)	-	73 (58.9)
Non-European Economic Area	22 (66.7)	29 (31.9)	-	51 (41.1)
**Suspects**				
1	18 (54.5)	56 (61.5)	-	74 (59.7)
2	10 (30.3)	19 (20.9)	-	29 (23.4)
3	3 (9.1)	5 (5.5)	-	8 (6.5)
4	1 (3.0)	2 (2.2)	-	3 (2.4)
≥5	1 (3.0)	9 (9.9)	-	10 (8.1)
**Concomitants**				
0	24 (72.7)	74 (81.3)	-	98 (79.0)
1	5 (15.2)	8 (8.8)	-	13 (10.5)
2	1 (3.0)	5 (5.5)	-	6 (4.8)
3	1 (3.0)	2 (2.2)	-	3 (2.4)
≥5	2 (6.1)	2 (2.2)	-	4 (3.2)
**Patients Over 18 Years**	**Atomoxetine**	**Methylphenidate**	**Combination**	**Overall**
	**(N = 183)**	**(N = 70)**	**(N = 8)**	**(N = 261)**
**Age**				
18–64 Years	182 (99.5)	66 (94.3)	8 (100)	256 (98.1)
65–85 Years	-	4 (5.7)	-	4 (1.5)
More Than 85 Years	1 (0.5)	-	-	1 (0.4)
**Sex**				
Female	155 (84.7)	26 (37.1)	7 (87.5)	188 (72.0)
Male	25 (13.7)	38 (54.3)	1 (12.5)	64 (24.5)
Not Specified	3 (1.6)	6 (8.6)	-	9 (3.4)
**Source**				
Healthcare Professional	173 (94.5)	58 (82.9)	8 (100)	239 (91.6)
Non-Healthcare Professional	10 (5.5)	11 (15.7)	-	21 (8.0)
Not Specified	-	1 (1.4)	-	1 (0.4)
**Country**				
European Economic Area	5 (2.7)	38 (54.3)	1 (12.5)	44 (16.9)
Non-European Economic Area	178 (97.3)	32 (45.7)	7 (87.5)	217 (83.1)
**Suspects**				
1	27 (14.8)	32 (45.7)	-	59 (22.6)
2	3 (1.6)	13 (18.6)	1 (12.5)	17 (6.5)
3	4 (2.2)	6 (8.6)	1 (12.5)	11 (4.2)
4	2 (1.1)	7 (10.0)	-	9 (3.4)
≥5	147 (80.3)	12 (17.1)	6 (75.0)	165 (63.2)
**Concomitants**				
0	96 (52.5)	41 (58.6)	2 (25.0)	139 (53.3)
1	14 (7.7)	9 (12.9)	-	23 (8.8)
2	10 (5.5)	6 (8.6)	-	16 (6.1)
3	8 (4.4)	2 (2.9)	-	10 (3.8)
4	12 (6.6)	4 (5.7)	-	16 (6.1)
≥5	43 (23.5)	8 (11.4)	6 (75.0)	57 (21.8)
**Age Not Specified**	**Atomoxetine**	**Methylphenidate**	**Combination**	**Overall**
	**(N = 16)**	**(N = 20)**	**(N = 0)**	**(N = 36)**
**Age**				
Not Specified	16 (100)	20 (100)	-	36 (100)
**Sex**				
Female	8 (50.0)	6 (30.0)	-	14 (38.9)
Male	7 (43.8)	10 (50.0)	-	17 (47.2)
Not Specified	1 (6.3)	4 (20.0)	-	5 (13.9)
**Source**				
Healthcare Professional	11 (68.8)	13 (65.0)	-	24 (66.7)
Non-Healthcare Professional	5 (31.3)	6 (30.0)	-	11 (30.6)
Not Specified	-	1 (5.0)	-	1 (2.8)
**Country**				
European Economic Area	8 (50.0)	9 (45.0)	-	17 (47.2)
Non-European Economic Area	8 (50.0)	11 (55.0)	-	19 (52.8)
**Suspects**				
1	10 (62.5)	13 (65.0)	-	23 (63.9)
2	2 (12.5)	4 (20.0)	-	6 (16.7)
3	1 (6.3)	1 (5.0)	-	2 (5.6)
≥5	3 (18.8)	2 (10.0)	-	5 (13.9)
**Concomitants**				
0	14 (87.5)	15 (75.0)	-	29 (80.6)
1	1 (6.3)	3 (15.0)	-	4 (11.1)
2	-	1 (5.0)	-	1 (2.8)
≥5	1 (6.3)	1 (5.0)	-	2 (5.6)

Data are expressed as N (%).

**Table 2:** The seriousness and outcomes of selected preferred terms within the system organ class (SOC) ‘hepatobiliary disorders’, which are considered to be related to the use of atomoxetine, methylphenidate, or a combination of the two, as reported in the EudraVigilance spontaneous reporting system between 1 January 2012 and 20 May 2025.

Patients Under 18 Years	Atomoxetine (N = 43)	Methylphenidate (N = 99)	Combination (N = 0)	Overall (N = 142)
**Seriousness**				
Not Serious	-	15 (15.2)	-	15 (10.6)
Serious	43 (100)	84 (84.8)	-	127 (89.4)
Life-Threatening	6 (14.0)	7 (7.1)	-	13 (9.2)
Disabling	-	6 (6.1)	-	6 (4.2)
Caused/Prolonged Hospitalisation	11 (25.6)	21 (21.2)	-	32 (22.5)
Other Medically Important Condition	26 (60.5)	50 (50.5)	-	76 (53.5)
**Outcome**				
Not Recovered/Not Resolved	2 (4.7)	22 (22.2)	-	24 (16.9)
Recovered/Resolved with Sequelae	-	3 (3.0)	-	3 (2.1)
Recovering/Resolving	6 (14.0)	9 (9.1)	-	15 (10.6)
Recovered/Resolved	20 (46.5)	31 (31.3)	-	51 (35.9)
Unknown	15 (34.9)	34 (34.3)	-	49 (34.5)
**Patients Over 18 years**	**Atomoxetine** **(N = 127)**	**Methylphenidate** **(N = 62)**	**Combination** **(N = 8)**	**Overall** **(N = 197)**
**Seriousness**				
Not Serious	4 (3.1)	6 (9.7)	-	10 (5.1)
Serious	123 (96.9)	56 (90.3)	8 (100)	187 (94.9)
Results in Death	85 (66.9)	-	-	85 (43.1)
Life-Threatening	2 (1.6)	5 (8.1)	-	7 (3.6)
Caused/Prolonged Hospitalisation	16 (12.6)	17 (27.4)	-	33 (16.8)
Other Medically Important Condition	20 (15.7)	34 (54.8)	8 (100)	62 (31.5)
**Outcome**				
Fatal	85 (66.9)	-	-	85 (43.1)
Not Recovered/Not Resolved	3 (2.4)	9 (14.5)	1 (12.5)	13 (6.6)
Recovered/Resolved with Sequelae	-	1 (1.6)	-	1 (0.5)
Recovering/Resolving	9 (7.1)	9 (14.5)	1 (12.5)	19 (9.6)
Recovered/Resolved	13 (10.2)	11 (17.7)	-	24 (12.2)
Unknown	17 (13.4)	32 (51.6)	6 (75.0)	55 (27.9)
**Age Not Specified**	**Atomoxetine** **(N = 16)**	**Methylphenidate** **(N = 20)**	**Combination** **(N = 0)**	**Overall** **(N = 36)**
**Seriousness**				
Not Serious	1 (6.3)	-	-	1 (2.8)
Serious	15 (93.2)	20 (100)	-	35 (97.2)
Results in Death	3 (18.8)	-	-	3 (8.3)
Life-Threatening	1 (6.3)	1 (5.0)	-	2 (5.6)
Caused/Prolonged Hospitalisation	2 (12.5)	3 (15.0)	-	5 (13.9)
Other Medically Important Condition	9 (56.3)	16 (80.0)	-	25 (69.4)
**Outcome**				
Fatal	3 (18.8)	-	-	3 (8.3)
Not Recovered/Not Resolved	1 (6.3)	-	-	1 (2.8)
Recovering/Resolving	1 (6.3)	2 (10.0)	-	3 (8.3)
Recovered/Resolved	2 (12.5)	3 (15.0)	-	5 (13.9)
Unknown	9 (56.3)	15 (75.0)	-	24 (66.7)

Data are expressed as N (%).

**Table 3:** Preferred terms for distributions within the system organ class ‘hepatobiliary disorders’ associated with atomoxetine, methylphenidate, or their combination. These are categorised by the type of hepatic damage and their seriousness, as reported in the EudraVigilance spontaneous reporting system between 1 January 2012 and 20 May 2025.

	Atomoxetine		Methylphenidate		Combination		Overall N (%)
	Serious	Not Serious	Serious	Not Serious	Serious	Not Serious	
**Hepatic failure, fibrosis, cirrhosis, and other liver damage-related conditions**							
Acute hepatic failure	1 (100)	-	4 (100)	-	-	-	5 (2.1)
Chronic hepatic failure	-	-	1 (100)	-	-	-	1 (0.4)
Drug-induced liver injury	102 (99)	1 (1)	46 (97.9)	1 (2.1)	6 (100)	-	156 (66.7)
Hepatic atrophy	-	-	1 (100)	-	-	-	1 (0.4)
Hepatic cirrhosis	3 (100)	-	1 (100)	-	1 (100)	-	5 (2.1)
Hepatic failure	6 (100)	-	7 (100)	-	-	-	13 (5.6)
Hepatic fibrosis	1 (100)	-	2 (100)	-	-	-	3 (1.3)
Hepatic necrosis	1 (100)	-	3 (100)	-	-	-	4 (1.7)
Hepatic steatosis	6 (100)	-	11 (78.6)	3 (21.4)	-	-	20 (8.5)
Hepatolenticular degeneration	1 (100)	-	1 (100)	-	-	-	2 (0.9)
Hepatocellular injury	-	-	1 (100)	-	-	-	1 (0.4)
Hepatotoxicity	3 (100)	-	4 (100)	-	-	-	7 (3)
Liver disorder	7 (87.5)	1 (12.5)	5 (71.4)	2 (28.6)	-	-	15 (6.4)
Subacute hepatic failure	1 (100)	-	-	-	-	-	1 (0.4)
**Hepatitis, non-infectious**							
Allergic hepatitis	-	-	1 (100)	-	-	-	1 (1.9)
Autoimmune hepatitis	1 (100)	-	4 (100)	-	-	-	5 (9.3)
Eosinophilic hepatitis	-	-	1 (100)	-	-	-	1 (1.9)
Hepatic cytolysis	-	-	5 (71.4)	2 (28.6)	-	-	7 (13)
Hepatitis	20 (100)	-	8 (88.9)	1 (11.1)	1 (100)	-	30 (55.6)
Hepatitis acute	2 (100)	-	3 (100)	-	-	-	5 (9.3)
Hepatitis fulminant	-	-	1 (100)	-	-	-	1 (1.9)
Hepatitis toxic	1 (100)	-	2 (100)	-	-	-	3 (5.6)
Steatohepatitis	-	-	1 (100)	-	-	-	1 (1.9)
**Liver-related investigations, signs and symptoms**							
Ascites	-	-	1 (100)	-	-	-	1 (1.1)
Hepatic function abnormal	3 (60)	2 (40)	13 (81.2)	3 (18.8)	-	-	21 (24.1)
Hepatomegaly	-	-	4 (80)	1 (20)	-	-	5 (5.7)
Hyperammonaemia	-	-	2 (100)	-	-	-	2 (2.3)
Hyperbilirubinaemia	2 (100)	-	7 (77.8)	2 (22.2)	-	-	11 (12.6)
Hypertransaminasaemia	-	-	-	1 (100)	-	-	1 (1.1)
Jaundice	15 (100)	-	17 (85)	3 (15)	-	-	35 (40.2)
Ocular icterus	3 (75)	1 (25)	3 (60)	2 (40)	-	-	9 (10.3)
Yellow skin	2 (100)	-	-	-	-	-	2 (2.3)

Data are expressed as N (%).

## Data Availability

The original contributions presented in this study are included in the article and Supplementary Materials. Further inquiries can be directed to the corresponding authors.

## Supplementary Materials

The following supporting information can be downloaded at: https://www.mdpi.com/article/10.3390/ph19071122/s1, Table S1: The distribution of terms that were considered to be unrelated to drug use and excluded from the main analysis. Table S2: Information Component (IC) and Associated Lower (IC025) and Upper (IC975) Limits.

## Abbreviations

The following abbreviations are used in this manuscript:

ADHD	Attention Deficit/Hyperactivity Disorder
ADHD-C	Attention Deficit/Hyperactivity Disorder—Combined Type
ADHD-H	Predominantly Hyperactive–Impulsive Type
ADHD-I	Predominantly Inattentive Type
ADR	Adverse Drug Reaction
AE	Adverse Event
ATX	Atomoxetine
CI	Confidence Interval
DAT	Dopamine
DILI	Drug-Induced Liver Injury
EEA	European Economica Area
EMA	European Medicines Agency
EV	EudraVigilance
HCP	Healthcare Professional
HLT	High Level Term
HLGT	High Level Group Term
ICSR	Individual Case Safety Report
LLT	Lowest Level Term
MedDRA	Medical Dictionary For Regulatory Activities
MPH	Methylphenidate
NET	Norepinephrine Transporter
PT	Preferred Term
READUS-PV	Reporting of a Disproportionality Analysis for Drug Safety Signal Detection Using ICSRs in Pharmacovigilance
ROR	Reporting Odds Ratio
ROS	Reactive Oxygen Species
SMQ	Standardised MedDRA Query
SOC	System Organ Class
