# Supplementary material for: Hepatic Safety Profile of Atomoxetine and Methylphenidate in Patients with ADHD: Disproportionality Analysis Using EudraVigilance Database Data
Source: Pharmaceuticals (Basel). 2026 Jul 21;19(7):1122. doi: 10.3390/ph19071122 (PMC13414661; doi:10.3390/ph19071122)
Supplement: Supplementary file 1 [file pharmaceuticals-19-01122-s001.zip › pharmaceuticals-4333321-supplementary.pdf]

Supplementary Material

Table S1. The distribution of terms that were considered to be unrelated to drug use and excluded from the main analysis.

	**Atomoxetine (N = 194)**	**Combination (N = 8)**	**Methylphenidate (N = 222)**	**Overall (N = 424)**
Cholecystitis	3 (1.5)	-	5 (2.3)	8 (1.9)
Cholelithiasis	3 (1.5)	-	13 (5.9)	16 (3.8)
Gallbladder disorder	1 (0.5)	-	1 (0.5)	2 (0.5)
Hepatic encephalopathy	1 (0.5)	-	-	1 (0.2)
Bile duct stone	-	-	2 (0.9)	2 (0.5)
Biliary colic	-	-	2 (0.9)	2 (0.5)
Biliary obstruction	-	-	1 (0.5)	1 (0.2)
Biloma	-	-	1 (0.5)	1 (0.2)
Cholangitis sclerosing	-	-	1 (0.5)	1 (0.2)
Cholestasis	-	-	3 (1.4)	3 (0.7)
Cholestasis of pregnancy	-	-	3 (1.4)	3 (0.7)
Gallbladder polyp	-	-	2 (0.9)	2 (0.5)
Gilbert's syndrome	-	-	1 (0.5)	1 (0.2)
Hepatic cyst	-	-	1 (0.5)	1 (0.2)
Hepatic infection	-	-	1 (0.5)	1 (0.2)
Hepatic mass	-	-	1 (0.5)	1 (0.2)
Hepatitis A	-	-	1 (0.5)	1 (0.2)
Hepatitis C	-	-	1 (0.5)	1 (0.2)
Jaundice cholestatic	-	-	1 (0.5)	1 (0.2)

Data are expressed as N (%).

Table S2. Information Component (IC) and Associated Lower (IC025) and Upper (IC975) Limits

ATX VS MPH

Drug	IC	Lower	Upper_
SOC Hepatic	0.819582	0.575167	0.996552
Drug-induced liver injury	1.414646	1.088374	1.650078
Hepatitis	1.374035	0.623639	1.898719
Jaundice	0.715097	-0.15588	1.316505

ATX F vs M

Drug	IC	Lower_CI	Upper_CI
SOC Hepatic	0.126569	-0.17813	0.346653
Drug-induced liver injury	0.515578	0.156161	0.7745
Hepatitis	0.140858	-0.79761	0.783975

MPH F vs M

Drug	IC	Lower_CI	Upper_CI
SOC Hepatic	-0.45298	-0.8815	-0.14549
Drug-induced liver injury	-1.24031	-3.00546	-0.16132
Hepatitis	0.50446	-1.05772	1.48906

ATX Under 17 vs Over 18

Drug	IC	Lower_CI	Upper_CI
SOC Hepatic	-0.23293	-0.7127	0.110212
Drug-induced liver injury	-1.94548	-3.15807	-1.14246
Hepatitis	-0.39722	-2.16237	0.68177
Jaundice	0.902865	-0.3995	1.754878

MPH Under 17 vs Over 18

Drug	IC	Lower_CI	Upper_CI
SOC Hepatic	0.337096	0.025688	0.561957
Drug-induced liver injury	0.517876	-0.11342	0.964151
Hepatitis	0.298534	-1.46661	1.377527
Jaundice	0.646737	-0.29173	1.289855

Psychiatric vs non pychiatric co-madication ATX

Drug	IC	Lower_CI	Upper_CI
SOC Hepatic	-0.61059	-1.05839	-0.28965
Drug-induced liver injury	-0.38715	-1.68951	0.464865
Hepatitis	-0.73338	-2.49853	0.345609
Jaundice	-1.14872	-2.05157	-0.52751

Psychiatric vs non pychiatric co-madication MPH

Drug	IC	Lower_CI	Upper_CI
SOC Hepatic	-0.70619	-1.1347	-0.3987
Hepatitis	-0.49108	-2.56044	0.715416
Jaundice	-1.19752	-2.96267	-0.11853
